# ECCDIA: an interactive web tool for the comprehensive analysis of clinical and survival data of esophageal cancer patients

**DOI:** 10.1186/s12885-020-07479-9

**Published:** 2020-10-12

**Authors:** Jingcheng Yang, Jun Shang, Qian Song, Zuyi Yang, Jianing Chen, Ying Yu, Leming Shi

**Affiliations:** 1 State Key Laboratory of Genetic Engineering, School of Life Sciences and Shanghai Cancer Hospital/Cancer Institute, Fudan University, Shanghai, 200438 China; 2 Department of Hematology and Oncology, The Affiliated Hospital of Hangzhou Normal University, Hangzhou, 310015 Zhejiang China; 3 Medical College of Soochow University, Suzhou, 215000 Jiangsu China; 4 Human Phenome Institute, Fudan University, Shanghai, 201203 China; 5 Fudan-Gospel Joint Research Center for Precision Medicine, Fudan University, Shanghai, 200438 China

**Keywords:** Esophageal cancer, Clinical data mining, Survival analysis, Nomogram, SEER

## Abstract

**Background:**

Esophageal cancer (EC) is considered as one of the deadliest malignancies with respect to incidence and mortality rate, and numerous risk factors may affect the prognosis of EC patients. For better understanding of the risk factors associated with the onset and prognosis of this malignancy, we develop an interactive web-based tool for the convenient analysis of clinical and survival characteristics of EC patients.

**Methods:**

The clinical data were obtained from The Surveillance, Epidemiology, and End Results (SEER) database. Seven analysis and visualization modules were built with Shiny.

**Results:**

The Esophageal Cancer Clinical Data Interactive Analysis (ECCDIA, http://webapps.3steps.cn/ECCDIA/) was developed to provide basic data analysis, visualization, survival analysis, and nomogram of the overall group and subgroups of 77,273 EC patients recorded in SEER. The basic data analysis modules contained distribution analysis of clinical factor ratios, Sankey plot analysis for relationships between clinical factors, and a map for visualizing the distribution of clinical factors. The survival analysis included Kaplan-Meier (K-M) analysis and Cox analysis for different subgroups of EC patients. The nomogram module enabled clinicians to precisely predict the survival probability of different subgroups of EC patients.

**Conclusion:**

ECCDIA provides clinicians with an interactive prediction and visualization tool for visualizing invaluable clinical and prognostic information of individual EC patients, further providing useful information for better understanding of esophageal cancer.

## Background

Esophageal cancer (EC) is considered as one of the most deadly malignancies with respect to incidence and mortality rate [1, 2]. Globally, EC was ranked the seventh for the incidence rate and the sixth for the mortality rate in 2018 [2]. Approximately 17,650 new cases of EC are expected to occur and 16,080 patients are predicted to die from esophageal cancer in the United States in 2019 [1]. Previous studies have revealed numerous risk factors that may affect the prognosis of EC patients [3–6]. Nevertheless, these studies have been outdated and unable to provide an interactive and continuously updated result for researchers and physicians.

Population-based studies have been widely utilized to predict patients’ survival outcomes and have played a significant role for clinical decision makers and for the recommendations of guidelines [7]. With the rise of interactive data analysis, there have been many tools to help us understand the molecular characteristics of EC, but there is still a lack of effective interactive web tools based on population statistics data of EC to help us fully understand the risk factors associated with the onset and prognosis of this malignancy. The Surveillance, Epidemiology, and End Results (SEER) database is an authoritative source for cancer statistics with comprehensive clinical and pathological information of cancer cases reported in the United States [8]. Based on SEER data, many studies have been conducted to explore the epidemic, clinicopathologic and prognostic characteristics of EC, and to examine numerous risk factors that might be affected [3–6], but no study provides an interactive visual analysis of all the characteristics of EC data based on the SEER database [9]. Moreover, because SEER data are updated annually, the value of statistical results published from these studies using “outdated data” is somehow limited, resulting in limited usage of these precious data. Clinicians who would like to obtain valuable and updated information on EC prognosis may find it hard to navigate the rich data in SEER in whichever way they want.

Herein, we developed a powerful user-friendly web-based platform called Esophageal Cancer Clinical Data Interactive Analysis (ECCDIA) using data on 77,273 EC patients in the SEER Program from 1975 to 2018. ECCDIA is able to provide on-line statistical analysis tools, including clinical factor ratio distribution by year, the Sankey plots presenting relationships between different clinical factors, the survival rate analysis, Kaplan-Meier (K-M) analysis, Cox analysis, and nomograms illustrating prediction of survival probability across subgroups. ECCDIA is an efficient and user-friendly tool to assist researchers and clinicians to understand esophageal cancer using interactive analysis tools which can help users quickly explore data using different visualization approaches. ECCDIA is freely accessible and available at http://webapps.3steps.cn/ECCDIA/.

## Methods

### Patient data

Patient data were retrieved from the SEER*Stat Version 8.3.5 database named Incidence-SEER 18 Regs Custom Data (with additional treatment fields), Nov 2018 Sub (1973–2016 varying) by using the case-listing session. The International Classification of Diseases for Oncology (ICD-O-3) was utilized to identify patients with esophageal squamous cell carcinoma (ESCC) (ICD-O-3 histologic type: 8050–8089) and esophageal adenocarcinoma (EAD) (ICD-O-3 histologic type: 8140–8389) [10]. The ICD-O-3 site codes for EC were C15.0, 15.1, 15.2, 15.3, 15.4, 15.5, 15.8, and 15.9. As the SEER database is a public one, there is no personal identification information for patients. Patients with diagnosed confirmation of positive histology and those of active follow up were included for analysis. Patients with unknown survival data were excluded. There were 77,273 patients for overall survival (OS) and 52,206 patients for cancer specific death (CSS).

### Construction of analysis modules

**Fig. 1:**
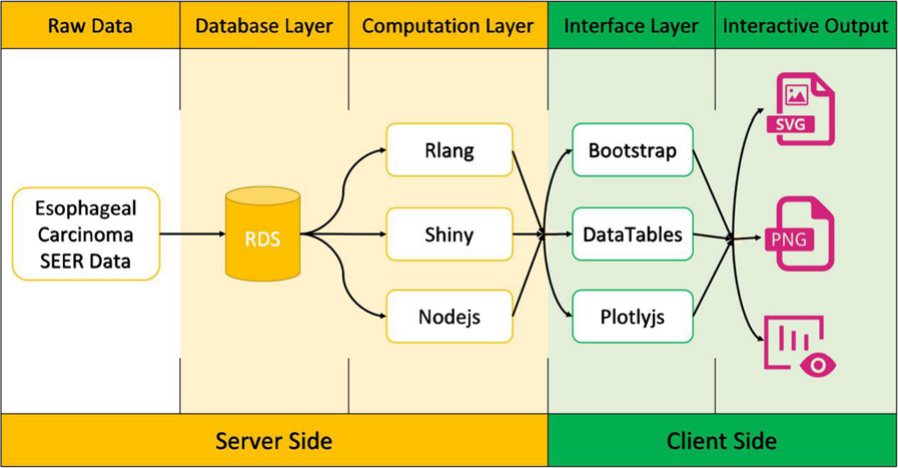
Framework of ECCDIA. Schema describing data processing and data visualization for the ECCDIA. Raw data contains information for 77,273 esophageal cancer (EC) patients. Computation layer contains survival analysis, Cox analysis and nomogram modules. Interactive output contains basic charts, such as bar plot, Sankey plot, line plot and map, etc.

ECCDIA is a web-based tool constructed with the Shiny framework. It contained seven interactive analysis modules written with R language (Fig. 1). Basic charts, such as bar plot, Sankey plot, line plot and map, were constructed with Plotly [11]. Cox and survival analysis were performed with R packages survival (v2.42–6) [12] and survminer (v0.4.3) [13]. Nomogram was constructed with R package rms (v5.1–2) [14].

## Results

### Data summary

Patients with unknown survival data were excluded. There were 77,273 patients including 58,668 males and 18,605 females for overall survival (OS) and 52,206 patients for cancer specific death (CSS). The histological type of 40,683 cases is esophageal adenocarcinoma (EAD), and the other 36,590 cases is esophageal squamous cell carcinoma (ESCC). Based on the 7th edition of AJCC, 3625, 3304, 5018 and 6287 patients were in stages I, II, III and IV, respectively.

### Modules of ECCDIA

**Fig. 2:**
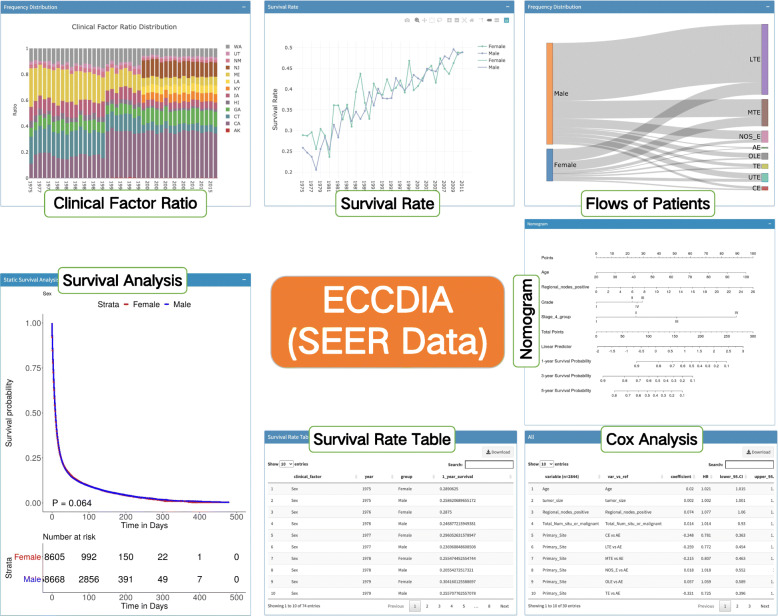
Overview of ECCDIA analysis modules. Clinical factor ratio aims to find out the trend of different clinical factor ratio distributions by year. Flows of patients provides users with a convenient and intuitive interface for the correlation of different clinical factors. Survival rate exhibits the changes of survival rate for clinical factors by year. The survival analysis module is used to compare the influence of clinical factors on OS and CSS in different subgroups of EC patients. Cox analysis module exhibits univariate and multivariate analysis of OS and CSS for different subgroups of EC patients. The Nomogram module predicts patients’ survival outcome for different subgroups of EC patients

ECCDIA is a modular interactive tool which mainly contains seven capabilities, including “Clinical Ratio” that analyzes clinical factor ratio distribution by year, “Sankey Plot” that demonstrates the relationship of frequency distribution between different clinical factors, “Survival Rate” that exhibits the changes of survival rate for clinical factors by year, “K-M Analysis” that displays survival curves of OS and CSS for clinical factors, “Cox Analysis” that exhibits univariate and multivariate analysis of OS and CSS for different subgroups of EC patients, “Nomogram” that predicts survival outcome for different subgroups of EC patients, and “Map” that exhibits the distribution of clinical factors in the form of a map of the United States (Fig. 2).

### Basic data analysis

The first module of ECCDIA aims to find out the trend of different clinical factor ratio distribution by year. As exhibited in Figure S1A, the incidence of EAD increased, while the incidence of ESCC decreased by year. To further investigate whether this trend existed in the subgroup of EC patients, the different subgroups of data could be chosen. Interestingly, we found that male and white patients had a similar trend as the whole group, but the female, black and API (Asian or Pacific Islander) patients did not demonstrate a significant trend change (Figure S1B-F). Additional fascinating results can be found by users using this module of ECCDIA.

The flows of patients’ module are to provide users with a convenient and intuitive interface for the correlation of different clinical factors. As demonstrated in Figure S2, most of the white patients were ESCC in 1975. In 1993, the proportion of EAD and ESCC was almost equal in white patients, whereas the majority of white patients were EAD in 2016. Users can perform interactive analysis in this module to find what they may be interested in. The last module of ECCDIA provides users with a map of the United States to show the distribution of clinical factors by state.

### Survival analysis

The survival analysis mainly contains three modules, including survival rate, K-M analysis, and Cox analysis. The survival rate module has the ability to show EC patients survival rate fluctuation by year. We showed in Figures S3A and S3B how to perform survival analysis quickly and easily. ECCDIA allows us to quickly find that there were clear differences in survival among different histopathological types and ethnicities. In Figure S4A, before 1989, there exhibited an obvious fluctuation of survival rate between EAD and ESCC. Nevertheless, EAD patients tended to have a consistently higher survival rate than ESCC patients after 1989.

The K-M analysis module provides intuitive figures for users who would like to compare the impact of clinical factors on OS and CSS in different subgroups of EC patients. For instance, Figure S4B-D exhibited a comparison of the impact of histologic types on patients’ OS. Regardless of male or female subgroup, EAD patients had a much better OS than ESCC patients.

The Cox analysis module demonstrates tables for the results of univariate and multivariate analysis of OS and CSS for different subgroups of EC patients. This module is also user-friendly and provides users with interactive tables.

### Nomogram

The nomogram module can precisely predict 1-year, 3-year, and 5-year survival probabilities of OS and CSS for all patients, ESCC patients, EAD patients, stages I-II patients, stages III-IV patients, and patients undergoing surgery plus chemotherapy and radiation therapy. For instance, for an EC patient who was 20 years old and had three positive regional nodes with grade I and stage I, the 1-year, 3-year, and 5-year survival probabilities were 96.82, 90.19, and 86.25%, respectively (Figure S5). At the same time, we show that there is a good agreement between the nomogram-based survival rates and the actual survival rate by using calibration plots (Figure S5C). Furthermore, the agreement between predicted and observed 1, 3, 5-year survival rates shown with calibration curves was verified with clinical data associated with the TCGA esophageal cancer data set (Figure S6).

### Map

“Map”, the last module of ECCDIA, provides users with a map of the United States to show the distribution of clinical factors by state. The distribution of the different survival rates is displayed in Data exploration and Interactive map sections. These functions can easily visualize the survival rate in different states for the EC patients (Table 1).

**Table 1:** The survival rates of esophageal cancer patients in different states

State	Num_Patients	Average_age	Average_tumor_size(mm)	1-year survival rate	2-year survival rate	3-year survival rate	4-year survival rate	5-year survival rate
Alaska	122	68.33	50.19	0.42	0.26	0.19	0.16	0.13
California	25,225	64.41	49.44	0.40	0.23	0.17	0.14	0.13
Connecticut	6832	65.74	48.40	0.41	0.26	0.19	0.16	0.14
Georgia	7832	67.88	45.13	0.48	0.31	0.24	0.20	0.17
Hawaii	1669	65.80	48.69	0.40	0.23	0.16	0.13	0.12
Iowa	4983	67.86	51.87	0.42	0.26	0.20	0.16	0.15
Kentucky	3484	68.03	50.49	0.43	0.27	0.21	0.18	0.16
Louisiana	3394	65.90	47.01	0.42	0.27	0.22	0.18	0.17
Michigan	7624	65.07	48.31	0.44	0.27	0.21	0.17	0.15
New Jersey	6762	67.49	53.91	0.39	0.22	0.17	0.13	0.11
New Mexico	1892	67.58	47.70	0.42	0.24	0.17	0.14	0.12
Utah	1503	67.27	55.90	0.34	0.18	0.13	0.10	0.08
Washington	5951	64.18	55.43	0.44	0.28	0.23	0.22	0.22

## Discussion

This study provides an interactive web tool that analyzes rich clinical and prognostic data of EC patients from the SEER database. Our tool is able to provide clinicians and clinical decision makers with useful information to make suitable treatment plan for EC patients with no need to refer to a large number of research papers.

The SEER database has such a large collection of cancer patients’ clinicopathologic and prognostic data so that it holds a great potential to conduct robust statistical mining to gain the most powerful and reliable survival prediction for cancer patients. However, previously published researches using the SEER database present analyses in only one aspect or the other and do not make full use of the comprehensive information in SEER. ECCDIA makes the most use of EC data of the SEER database and presents these data in a user-friendly interactive interface with no need to grasp computational programming skills. It can easily exhibit the clinicopathologic and prognostic analysis for a variety of subgroups of EC patients.

To the best of our knowledge, the online tool ECCDIA is the first such system that demonstrates the most comprehensive integrative analysis of clinical data with the full utilization of EC data in the SEER database. More importantly, to facilitate clinical use of this online tool, nomograms predicting the prognosis of different subgroups of EC patients are provided are provided by ECCDIA. Using ECCDIA, clinicians can immediately obtain the survival probability of patients by simply inputting the values of clinical factors, which helps them make the right decision for EC patients.

There are already many good online tools for esophageal cancer. For example, both OSescc and OSeac are great tools that use gene expression data of esophageal cancer patients in public databases to quickly query the correlation between the expression level of a gene and patient prognosis [15, 16]. Briefly, compared with OSescc and or OSeac, ECCDIA is committed to creating dynamic interactive visualization tools to explore the epidemiological characteristics of esophageal cancer in the SEER database. In addition to survival analysis, ECCDIA can also dynamically and interactively display the epidemiological characteristics of esophageal cancer patients spanning 20 years. Both gene expression data and epidemiological characteristics can provide complementary information for better understanding esophageal cancer.

Some limitations of ECCDIA need to be mentioned. ECCDIA does not integrate the molecular or genetic data of EC patients with their clinical data, since the SEER database only provides the clinical data of cancer patients. In addition, some treatment biases are present. Therefore, additional future work is needed by combining the data in the SEER database with other publicly available databases.

Nevertheless, ECCDIA is the first interactive web tool to assess the largest clinical and prognostic data of EC patients, which will become an invaluable resource for clinical guideline of EC. Besides, ECCDIA will be updated to embrace the newest data released by SEER.

## Conclusion

The Esophageal Cancer Clinical Data Interactive Analysis (ECCDIA, http://webapps.3steps.cn/ECCDIA/) is the first interactive prediction and visualization web tool to assess the largest clinical and prognostic data of EC patients from the SEER database, further increasing the assessment of clinical guidelines for EC. Furthermore, ECCDIA will be regularly updated to embrace the newest data to be released by SEER.

## Supplementary information

**Additional file 1: Figure S1.** Histologic type ratio distribution by year. **Figure S2.** The patient flows between histologic type and race. **Figure S3.** Survival analysis example. **Figure S4.** The relationship between histologic type and survival. **Figure S5.** Survival rate prediction. **Figure S6.** Survival prediction external verification.

## Data Availability

The datasets generated and/or analyzed during the current study are available in the clinico-omics/ECCDIA repository, https://github.com/clinico-omics/ECCDIA.

## Abbreviations

EC: Esophageal cancer
SEER: The Surveillance, Epidemiology, and End Results
KM: Kaplan-Meier
ECCDIA: Esophageal Cancer Clinical Data Interactive Analysis
ICD-O-3: International Classification of Diseases for Oncology
ESCC: Esophageal squamous cell carcinoma
EAD: Esophageal adenocarcinoma
OS: Overall survival
CSS: Cancer specific death
