# Supplementary material for: ECCDIA: an interactive web tool for the comprehensive analysis of clinical and survival data of esophageal cancer patients
Source: BMC Cancer. 2020 Oct 12;20:985. doi: 10.1186/s12885-020-07479-9 (PMC7552344; doi:10.1186/s12885-020-07479-9)

**Figure S1 Histologic type ratio distribution by year**

(A) AD and SCC distribution in all cohort. (B-F) AD and SCC distribution in Female, Male, White, Black, and API subgroups.


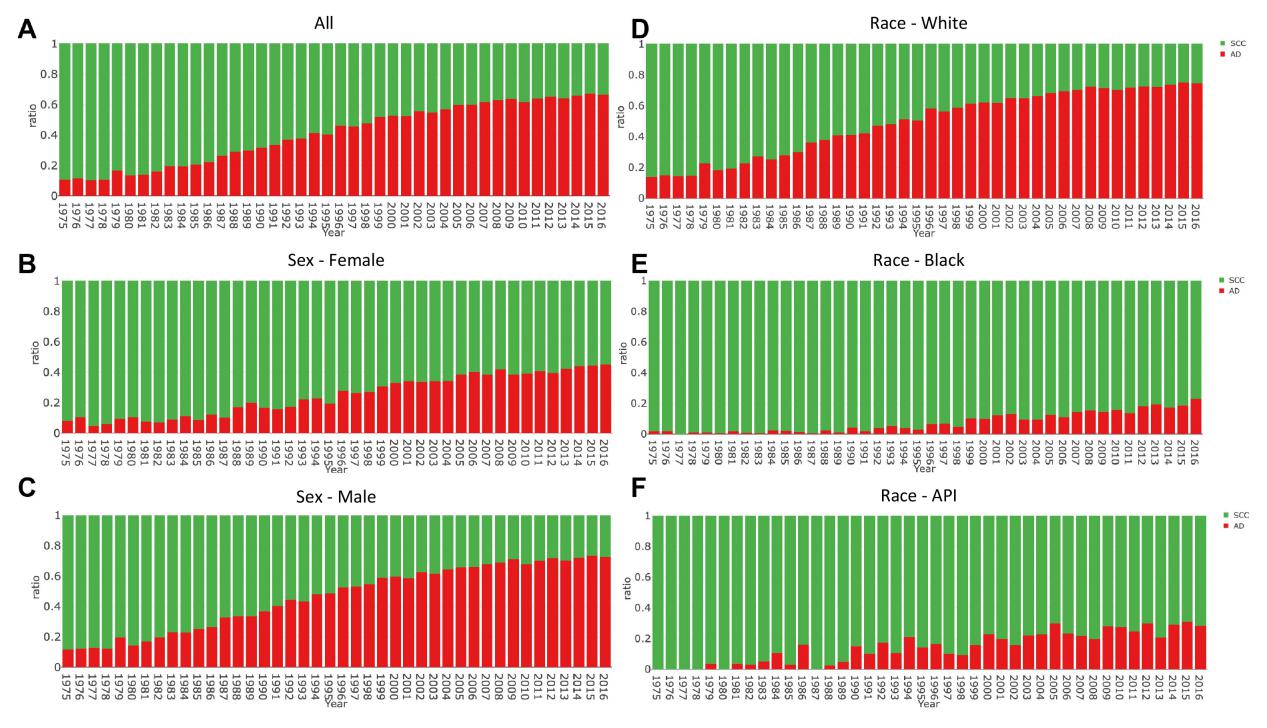


**Figure S2 The patient flows between histologic type and race**

(A) all cohort. (B-D) 1975, 1993 and 2016 subgroups.


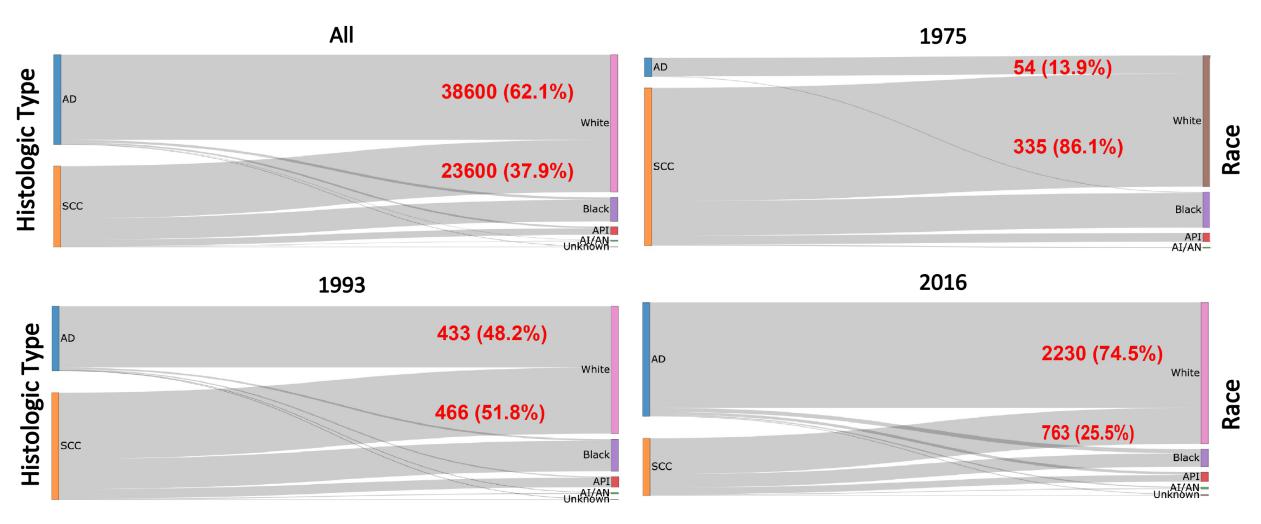


**Figure S3 Survival analysis example**

(A-B) ECCDIA was used to quickly and conveniently analyze the survival of patients with different histopathological types and races. There were clear differences in survival among different histopathological types and ethnicities.

**
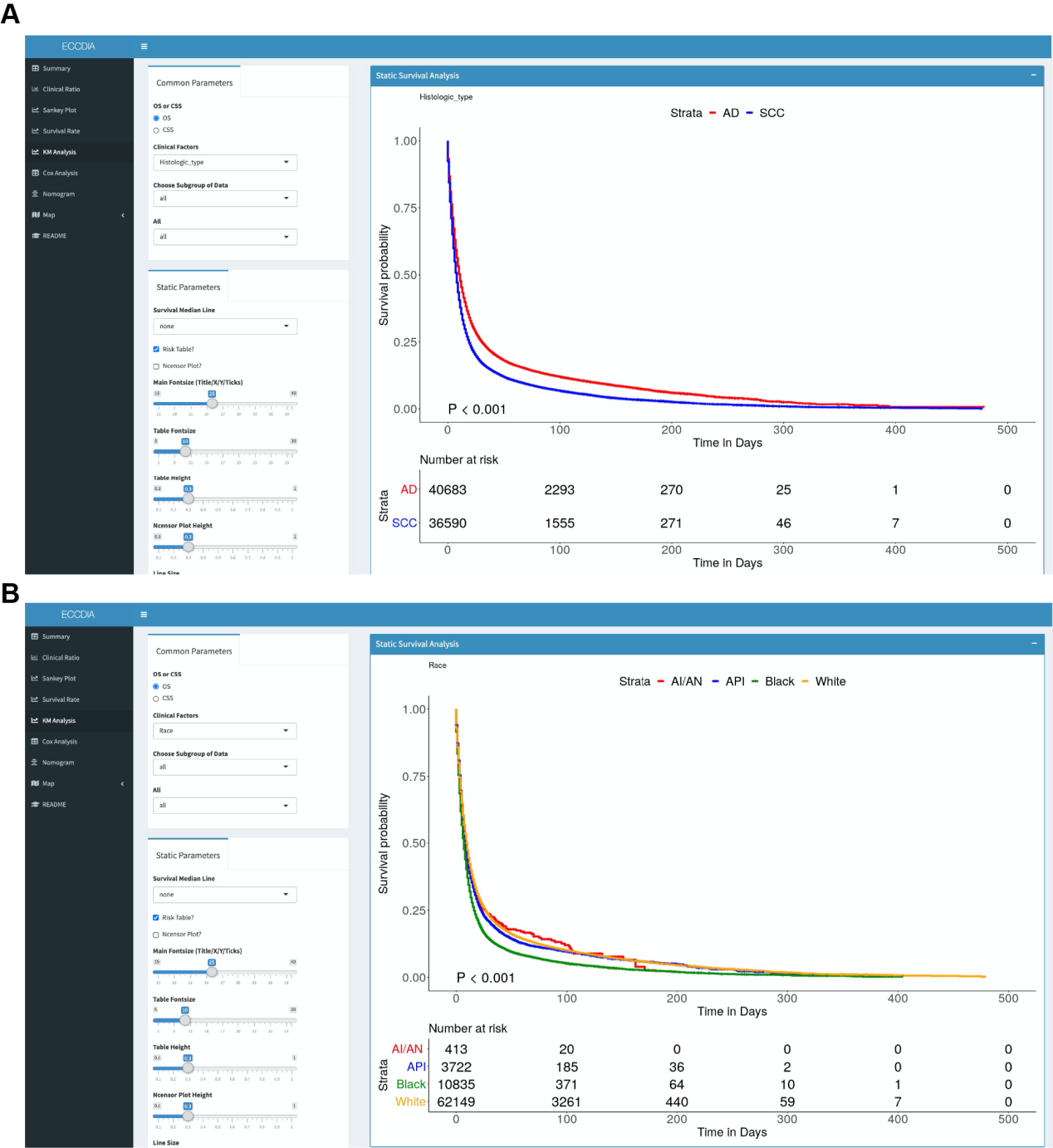
**

**Figure S4 The relationship between histologic type and survival**

(A) AD and SCC survival rate fluctuation by year. (B) survival analysis in all cohort. (C-D) survival analysis in Male and Female subgroups.

**
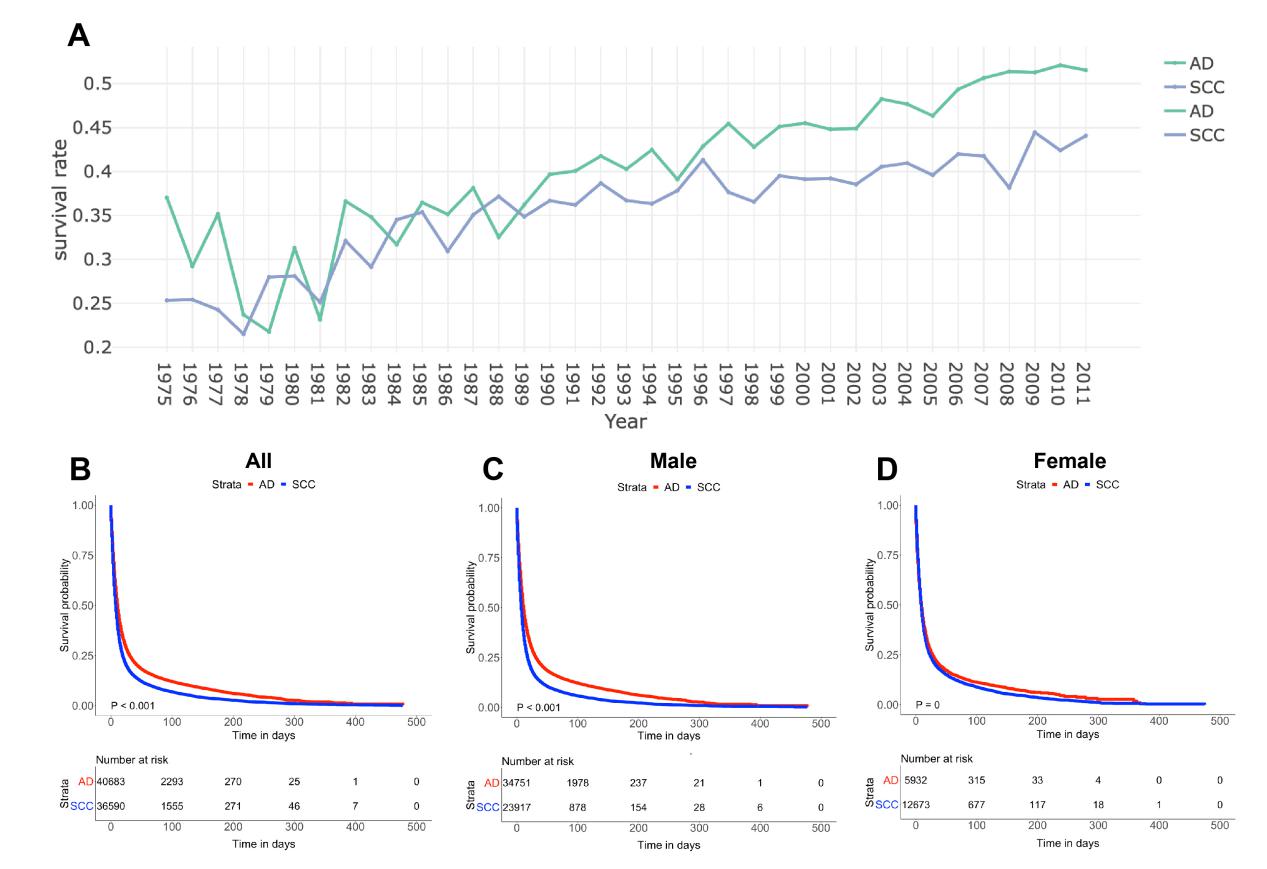
**

**Figure S5 Survival rate prediction**

(A) Clinical factors used to predict 1, 3, 5-year survival rate. (B) Clinical factors including age, regional nodes positive, grade and stage were used to construct nomogram in the train cohort. (C) The agreement between predicted and observed 1, 3, 5-year survival rates shown with calibration curves.


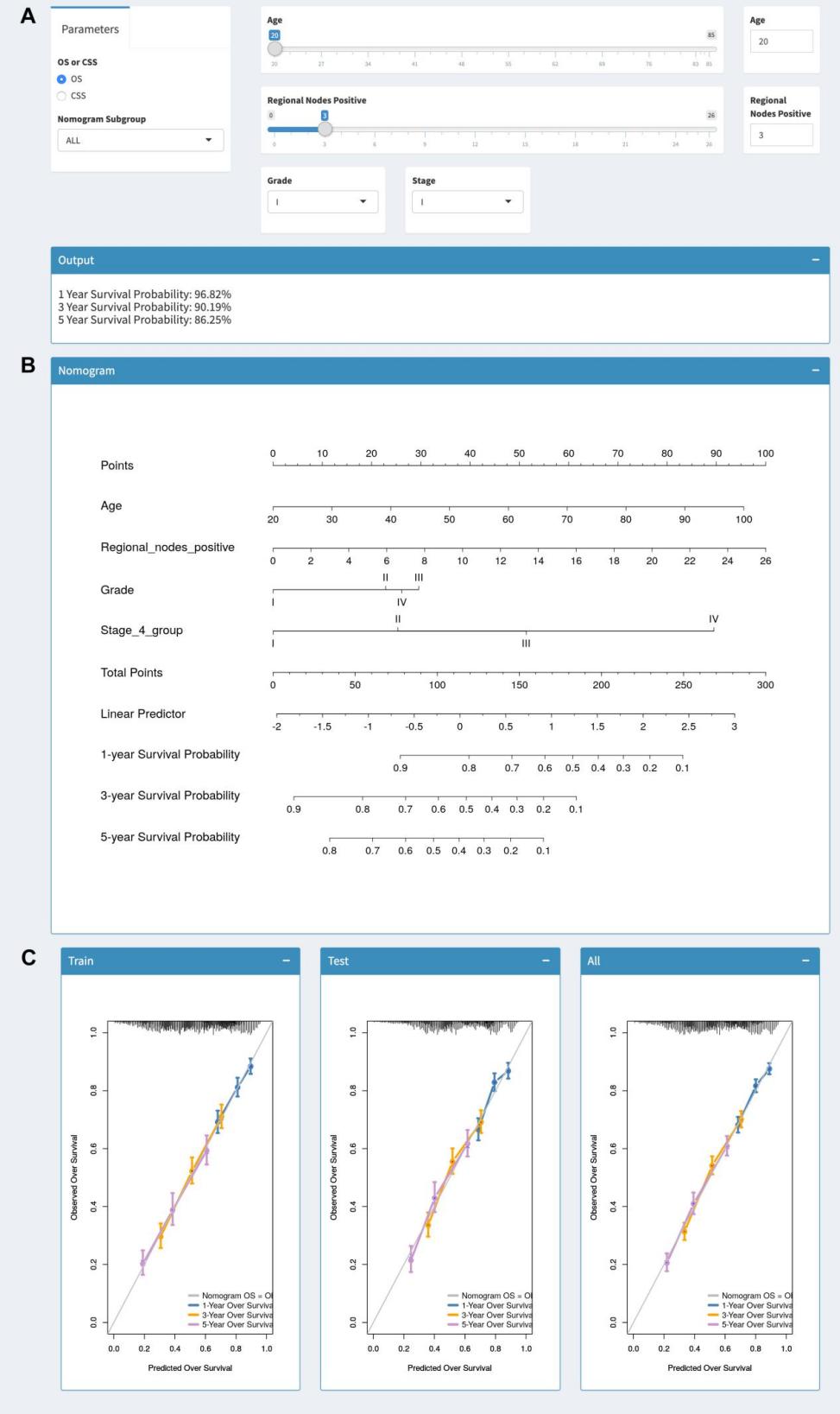


**Figure S6 Survival prediction external verification**

The agreement between predicted and observed 1, 3, 5-year survival rates shown with calibration curves was verified in the TCGA data set.


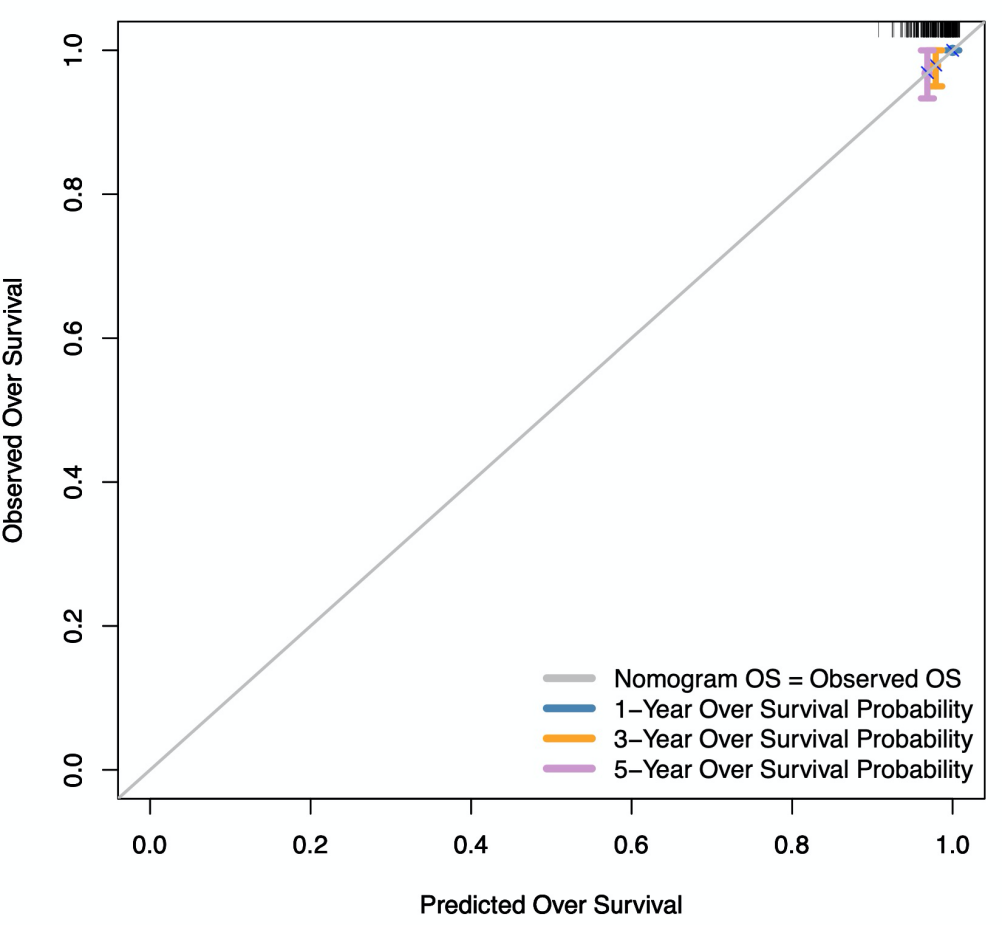

Supplement: Supplementary file 1 — Additional file 1: Figure S1. Histologic type ratio distribution by year. Figure S2. The patient flows between histologic type and race. Figure S3. Survival analysis example. Figure S4. The relationship between histologic type and survival. Figure S5. Survival rate prediction. Figure S6. Survival prediction external verification. [file 12885_2020_7479_MOESM1_ESM.docx]
